# Analysis of Mechanical Characteristics of the Swing Angle Milling Head of a Heavy Computer Numerical Control Milling Machine and Research on the Light Weight of a Gimbal

**DOI:** 10.3390/ma17020324

**Published:** 2024-01-09

**Authors:** Youzheng Cui, Chengxin Liu, Haijing Mu, Hui Jiang, Fengxia Xu, Yinfeng Liu, Qingming Hu

**Affiliations:** 1 School of Mechanical and Electronic Engineering, Qiqihar University, Qiqihar 161006, China; liuchengxin1088@163.com (C.L.); muhaijinghust@163.com (H.M.); 01541@qqhru.edu.cn (F.X.); 03313@qqhru.edu.cn (Y.L.); 2 The Engineering Technology Research Center for Precision Manufacturing Equipment and Industrial Perception of Heilongjiang Province, Qiqihar 161006, China; 3 The Collaborative Innovation Center for Intelligent Manufacturing Equipment Industrialization, Qiqihar 161006, China; 4 Qiqihar Heavy CNC Equipment Co., Ltd., Qiqihar 161005, China; qzsk_jh@163.com; 5 School of Mechatronics Engineering, Harbin Institute of Technology, Harbin 150001, China

**Keywords:** heavy milling machines, milling head, finite-element analysis, universal frame, topological optimization

## Abstract

As the key component of a five-axis CNC planer-type milling machine, the integral mechanical property of the A/C swing angle milling head directly affects the machining accuracy and stability of the milling machine. Taking the mechanical A/C swing-angle milling head of a five-axis numerical-control gantry milling machine as the research object, the stress deformation characteristics and natural frequency of the swing-angle milling head under actual working conditions were studied using finite-element analysis. Based on the analytical results, it was determined that the cardan frame, with its large mass proportion and strong rigidity of the whole milling head, is the object to be optimized. The topological optimization of the cardan frame, in which achieving the minimum flexibility was the optimization objective, was carried out to determine the quality reduction area. By comparing the simulation results of the cardan frames of three different rib plate structures, it was shown that the cardan frame performance of the ten-type rib plate structure was optimal. The analytical results showed that, when the cardan frame met the design requirements for stiffness and strength, the mass after optimization was reduced by 13.67% compared with the mass before optimization, the first-order natural frequency was increased by 7.9%, and the maximum response amplitude was reduced in all directions to avoid resonance, which was beneficial to the improvement of the dynamic characteristics of the whole machine. At the same time, the rationality and effectiveness of the lightweight design method of the cardan frame were verified, which has strong engineering practicality. The research results provide an important theoretical basis for the optimization of other machine tool gimbals and have important practical significance and application value.

## 1. Introduction

Topological optimization is widely used in machinery, aerospace and other fields due to its high design freedom and wider design space [1]. The double-swing angle milling head is one of the core components of the five-axis CNC planer milling machine, and its dynamic and static characteristics directly affect the overall working performance of the machine tool. Research on key parts of the machine tool via topological optimization is an important means for lightweight research [2,3]. As the cardan frame is the heaviest part of the swing angle milling head, the realization of its lightweight design has an important impact on improving the machining accuracy of the whole swing angle milling head, thus having important practical significance.

Scholars at home and abroad have carried out extensive research on many key parts of machine tools. Some scholars [4] have carried out optimization research on the structure of the swing angle milling head’s cardan frame. The results before and after optimization have shown that the new cardan frame has a good optimization effect while the rigidity and strength meet the design requirements. Francesco A [5] et al. realized the lightweight design of machine tool components using mixed lightweight materials to improve the machining performance. Raz K [6] et al. optimized the milling head’s topology, and the results showed that the milling head mass was reduced and the rigidity was improved. TriM J et al. [7] studied the sliding table of a three-axis vertical milling machine to reduce the energy consumption of the machine tool and successfully realized a lightweight design. Shindo R et al. achieved a lightweight machine tool structure by combining topological optimization and shape optimization methods on the basis of not following the basic design principles for the non-optimal design problem of the machine tool (i.e., a bending structure that cannot be avoided). Compared with the traditional design, the optimization method proposed in the study greatly improved the machine tool’s natural frequency and efficiency [8]. Li Congbo [9] and others comprehensively considered the energy consumption and dynamic and static performance of the machine tool. On the premise of ensuring both the static and dynamic performance, they not only reduced the energy consumption of the optimized structure, but also achieved a lightweight structural design. Petra L [10] et al. designed and studied the moving parts of the gantry-type CNC machine tool and performed a topological optimization of these moving elements. Under the premise of maintaining the same rigidity, compared with the original structural design, its stability was guaranteed, although the quality was reduced by about 25%. TRIEN et al. [11] proposed a lightweight design of the machine tool using a genetic algorithm, and the results showed that the energy consumption of the machine tool was reduced and that the rigidity met the requirements. Ko and Lee et al. [12] focused on studying the constrained stiffness optimization problem, considering the maximum allowable displacement. Based on genetic algorithms, they conducted optimization research on the size and weight of five-axis machine tools. Abdelwahab et al. [13] used topology optimization methods and AM manufacturing processes to reduce the weight of the machine tool box structure, while meeting the design requirements in terms of stiffness and strength. Kim, Seung-Gi et al. [14] realized the optimal design of important structures such as the machine tool’s ram and column parts via the topological optimization method, and they achieved good rigidity and weight. In order to improve the performance of the planer milling machine, BES et al. [15] studied the milling machine through the analytic hierarchy process. The research showed that the method proposed in the article can better realize the selection and structural optimization of the gantry. The optimized designs presented by the above scholars based on finite-element simulation analyses demonstrate the advantages of lightweight machine tool parts and also provide a reference for follow-up research.

The combination of finite-element analysis and topological optimization methods can effectively achieve the goal of a lightweight design for machine tool components. At the same time, based on the topology optimization results and the production process, different rib plate structures can be introduced into the reduced-quality area of machine tool components to improve their static and dynamic performance. For example, Dimitrov et al. [16] improved the stiffness of a structure by welding or casting reinforcement ribs, and they achieved their goal of removing unnecessary materials on the fuselage by using finite-element analysis and topological optimization methods. Tzu et al. [17] used a combination of finite-element methods and topological optimization methods to analyze and optimize the operating structure of CNC machine tools in order to propose better optimization design solutions for key components of the machine tool. The work of Ullah et al. [18] is crucial for the design and improvement of precision machinery based on static and dynamic analyses. By comparing the data with the results of the finite-element analysis (FEA), the static and dynamic characteristics, accuracy and efficiency of the finite-element model were verified. On the basis of improving the dynamic characteristics of machine tools, scholars such as Guido Stoppler have optimized the topology of the crossbeam structure to achieve a lightweight performance [19]. Hu Shijun et al. [20] proposed an “X” rib plate as a new beam with a good performance, verifying its performance by comparing the static and dynamic performance of the commonly used five-rib plate structures. The results showed that the performance of the optimized machine was improved to different degrees and that the beam mass was reduced by 2.1%. Bakker C et al. [21] proposed a method for optimizing the stiffened plate and its structural layout to improve the performance of the stiffened plate shell. Jiang Shufeng et al. [22] designed and researched the DVT250 machine tool crossbeam, and their results show that the diamond rib plate structure, as the optimized crossbeam structure, has the best lightweight effect. Liu Chengying et al. [23] optimized the standing column of the machine tool by means of multiple optimization methods and selected the design scheme of a W-shaped plate rib with optimal parameters to improve the natural frequency of the machine tool and realize its lightweight design. Based on the above literature analysis, it can be concluded that the combination of finite-element analysis and topology optimization, combined with the introduction of a rib plate structure, can provide theoretical reference and methodological support for the optimization and lightweight design of the swing angle milling head structure studied in this paper.

Based on the research and analysis of the above literature, it can be seen that more research has been carried out on the spindle boxes, rib plates and beams of machine tools, while less research has been carried out on the gimbals of swing angle milling heads. Furthermore, topology optimization has been mainly carried out for the gimbals of direct drive swing angle milling heads. At the same time, the gimbal accounts for a large proportion of the quality of the whole swing angle milling head, and it is also the basis for the installation of other parts. Therefore, it is very necessary to perform lightweight research on the gimbal of the swing angle milling head. In this paper, the mechanical A/C swing angle milling head of a five-axis CNC gantry milling machine is taken as the research object. Based on static and modal analyses, the gimbal structure with the strongest rigidity and the largest mass proportion of the milling head is identified as the object to be optimized. The topology optimization research is carried out for the case of less deformation (i.e.,higher strength) of the upper end of the gimbal, and a comparative analysis of the gimbal with three kinds of rib plate structures is carried out. It is found that the performance of the ten-type rib plate structure is better. After optimization, under the premise of ensuring that the stiffness and strength of the optimized universal joint meet the design requirements, the purpose of achieving a lightweight design is fulfilled. This research combines the topology optimization method with the rib plate structure, which can not only reduce the mass of the swing angle milling head, but also improve the dynamic characteristics of the whole machine. It has an important theoretical basis and practical significance for the optimization design of subsequent structures and for improving machine performance.

## 2. A/C Swing Angle Milling Head Structural Analysis and Finite-Element Analysis Pretreatment

### 2.1. Structural Analysis, Simplification and Mesh Generation of the Milling Head Model

**Figure 1:**
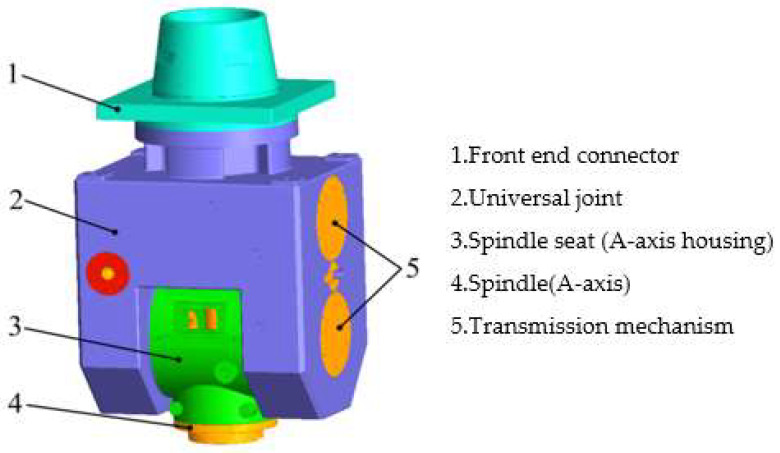
Model diagram of a mechanical A/C swing angle milling head.

This article takes the swing angle milling head of a five-axis CNC gantry milling machine as the research object. Its structure is composed of a transmission mechanism, a universal frame, an A-axis (main shaft), the A-axis housing (main shaft seat), front-end connectors and other parts. The overall structure is shown in Figure 1. The overall size of the A/C double swing angle milling head is 1550 mm × 830 mm × 930 mm.

**Figure 2:**
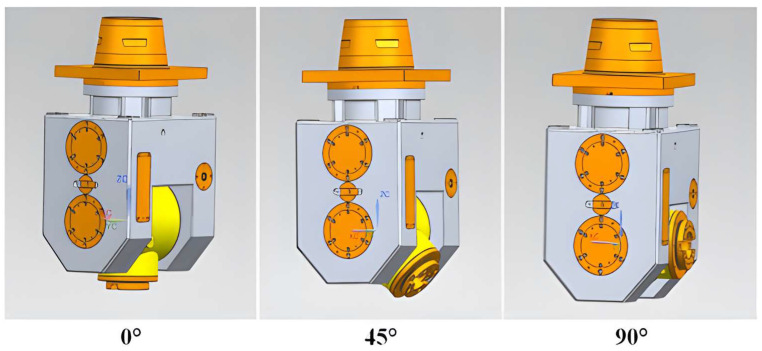
Three angles of the mechanical A/C swing angle milling head.

In order to study the static characteristics of the swing angle milling head under different working conditions, three typical milling head angles (i.e., 0°, 45° and 90°) are selected for comparative analysis, as shown in Figure 2.

**Figure 3:**
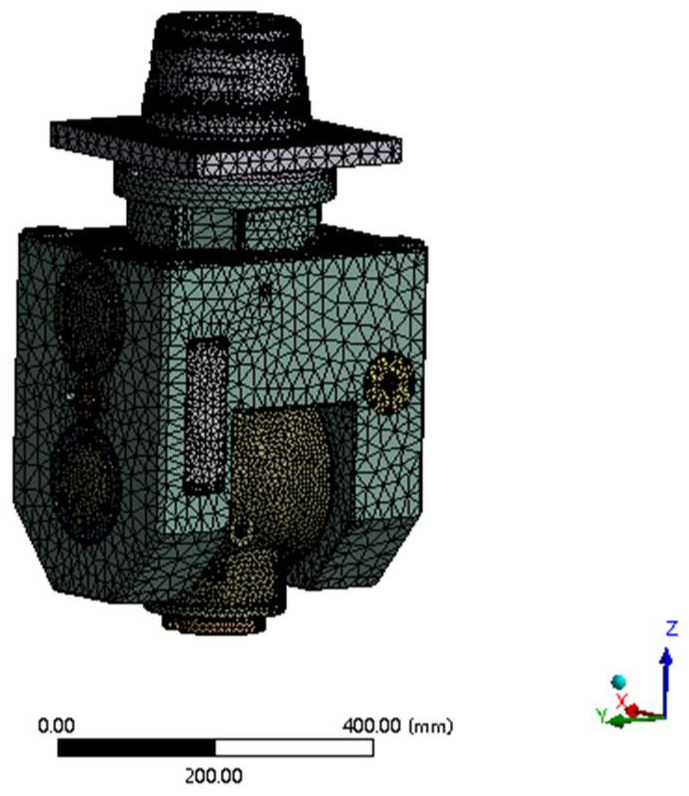
Finite-element mesh generation of a mechanical A/C swing angle milling head.

Due to the complexity of the internal structure of the A/C swing angle milling head, this paper simplifies the relatively minor process features in the digital model of the swing angle milling head, such as the chamfer, fine hole, undercut, oil channel, etc., in order to reduce the calculation time of the finite-element analysis. Then, the simplified swing angle milling head model is imported into ANSYS Workbench (2021 R1, ANSYS company, Pittsburgh, PA, USA) for mesh generation. In order to obtain ideal calculation results, the A/C swing angle milling head model is discretized by using the automatic mesh generation method, and the number of nodes and elements in the model is 976,796 and 572,014, respectively. The finite-element mesh generation model of the swing angle milling head is shown in Figure 3.

### 2.2. Material Properties of the Milling Head

In this paper, the material used for the box and rotary mechanism of the A/C swing angle milling head is ductile cast QT600-3, and the material of all shafting components is 38CrMoAl. Among the components, the gear and bearing inside the milling head are made of alloy structural steel. See Table 1 for the specific parameters of the materials used in the milling head simulation.

### 2.3. Load and Boundary Conditions of Swing Angle Milling Head

The external loads to be borne by the A/C swing angle milling head during the machining process mainly include the milling force and gravity. Since the gravitational load can be applied as set parameters in the simulation software and directly added for use, it is only necessary to calculate the cutting force of the milling head according to the empirical formula. Taking the A/C swing angle milling head assembled on a heavy CNC planer-type milling machine as an example, tungsten steel (a hard alloy) is selected as the cutter material and bearing steel is selected as the machined material. The symmetric end milling method is adopted, and the total milling force of the mechanical A/C swing angle milling head of the five-axis CNC planer-type milling machine can be calculated according to the following empirical formula [24]:

(1) $$\left\{\begin{array}{c} F_{C}=C_{FC}\times Z\times D^{-1.1}\times {a_{e}}^{1.1}\times a_{p}^{0.95}\times f_{z}^{0.8}\times K_{FC}\\ K_{FC}={\left(\frac{\sigma_{b}}{637}\right)}^{0.3} \end{array}\right.$$

In the formula, *F_C_* is the total milling force, *C_FC_* is the milling force coefficient, *K_FC_* is the correction coefficient, *D* is the milling cutter diameter, *Z* is the number of milling cutter teeth, *f_z_* is the feed rate per tooth, *a_e_* is the cutting width, *a_p_* is the number of back tooth cutters and $\sigma_{b}$ is the tensile strength. By substituting relevant milling parameters in Table 2, the total milling force is 1184.01. The components of the milling force acting on a mechanical A/C swing angle milling head in all directions are as follows: transverse feed force *F_e_*, longitudinal feed force *F_h_* and vertical feed force *F_v_*. The geometric relationship with the total milling force is set as follows:

(2) $$\left\{\begin{array}{c} F_{e}=0.9F_{c}\\ F_{h}=0.3F_{c}\\ F_{v}=0.5F_{c} \end{array}\right.$$

After calculation, the total milling force is decomposed into three milling forces along the moving direction of the workpiece, namely, the transverse feed force $F_{e}=1065.6\;N$ , the longitudinal feed force $F_{h}=355.2\;N$ and the vertical feed force $F_{v}=592\;N$ .

**Figure 4:**
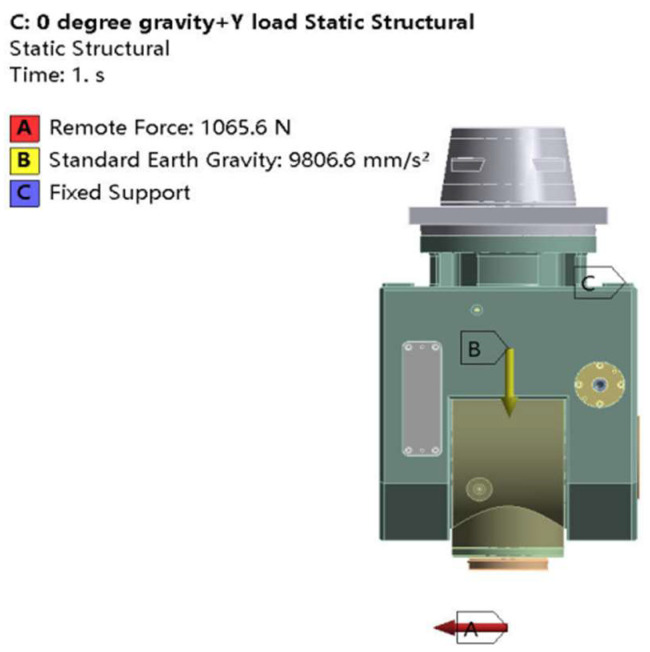
Restraint and load model at 0° for the A-axis of the milling head.

Before conducting static analysis, it is necessary to add loads and constraints. A fixed constraint is applied to the upper-end face of the swing angle milling head, and then the load step method in ANSYS is used to add a standard gravitational load to the connection surface between the swing angle milling head and the slider. The milling force is applied in the form of a remote force in the model, with the point of action set at a distance of 100 mm from the tip of the swing angle milling head [25]. The deformation caused by the longitudinal feed force *F_h_* and vertical feed force *F_v_* is relatively small and has little influence on the machining accuracy of the milling head, and thus can be ignored. Therefore, in this paper, the effect of the cross-feed force Fe on milling head deformation is selected. An infeed force Fe is added to the model in the form of a remote force. Figure 4 shows the constraints and loadings of the milling head under gravitational and milling forces in the Y direction when the milling head’s A-axis is at 0°. Note that gravitational and milling forces need to be loaded separately when the load is applied.

## 3. Analysis of Static and Dynamic Characteristics

### 3.1. Static Analysis of Swing Angle Milling Head

**Figure 5:**
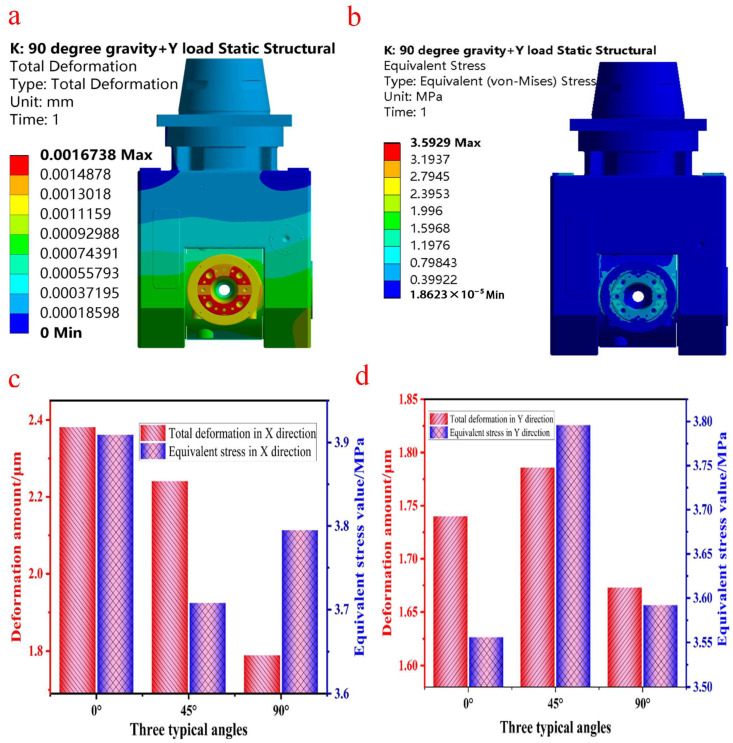
Results diagram of different angles of the swing angle milling head under the action of gravity and milling force in the (X, Y) direction. (**a**) Deformation cloud map at an angle of 90° under gravity and Y load. (**b**) Stress cloud map at an angle of 90° under gravity and Y load. (**c**) Stress and deformation values at different angles under gravity and X load. (**d**) Stress and deformation values at different angles under gravity and Y load.

Since the deformation of the milling head structure caused by the gravitational load can be eliminated, only the static simulation analysis results of the milling force deformation are given here. The results under the combined action of the output gravity and milling force obtained through finite-element analysis are shown in Figure 5. Through the simulation analysis of milling head angles of 0°, 45° and 90°, it can be seen that the maximum deformation areas of the three angles are distributed in the same area in the milling head, that is, the connecting tool shank. The distribution of the maximum stress is also the same, except it is distributed in the cutter head area of the milling head’s A-axis, which is consistent with the actual working conditions, indicating that the analysis’s conclusion is reasonable and effective. Therefore, in this paper, the limit angle under working conditions (i.e., the A-axis at 90°) is selected based on three typical angles as examples, and the analytical results are shown in Figure 5a,b.

According to the deformation cloud chart in Figure 5a, the maximum deformation area of the A-axis of the swing angle milling head is mainly concentrated at the connecting tool handle under the action of the milling force in the Y direction, while the area next to the maximum deformation is located at the bottom of both sides of the milling head, which has room for degradation. At the same time, the upper part of the milling head has less deformation and a better stiffness, and is thus the key optimization area. According to the equivalent stress nephogram in Figure 5b, the maximum stress of the milling head is 3.59 Mpa, which is far lower than the strength limit of the milling head material. Considering the influence of the A-axis swing angle further, the deformation at different angles is analyzed. According to the stress and deformation values of the X-load and Y-load in Figure 5c,d, the total deformation and equivalent stress of the A-axis of the swing angle milling head fluctuate at different angles. The total deformation fluctuates most obviously under the action of the X-axis milling force: its fluctuation value reaches 0.592 μm. The equivalent stress value under the action of the Y-axis milling force fluctuates greatly, and its fluctuation value reaches 0.24 Mpa. When the milling head’s A-axis is at 0° under the action of the X-direction milling force, the maximum deformation is 2.381 μm. In addition, when the milling head’s A-axis is set at different angles, the maximum deformation area is also concentrated on the connecting tool handle. The maximum stress of the milling head under different angles occurs in the A-axis cutter head area, which is consistent with the actual working conditions. This shows that the area with better stiffness and less stress (the upper part of the milling head) can be optimized to prepare for the subsequent lightweight design.

### 3.2. Modal Analysis of Swing Angle Milling Head

**Figure 6:**
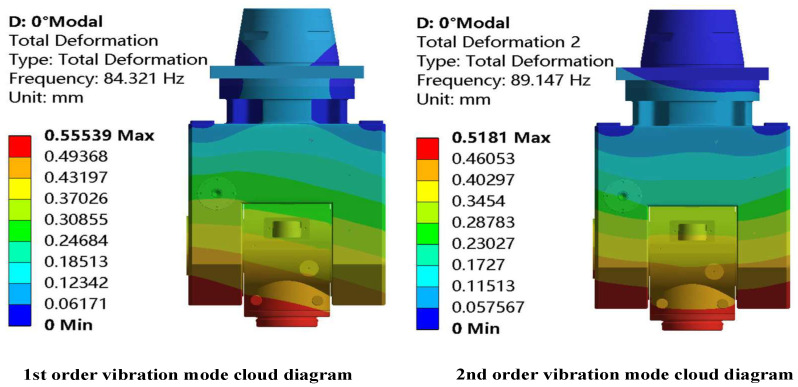
The first two vibration modes of the mechanical A/C double swing angle milling head at an angle of 0°.

ANSYS Workbench was used to conduct modal analysis on the double swing angle milling head of the machine tool. The dynamic performance of the swing angle milling head is basically determined by the low-order natural frequency [26], so only the first two modes of the swing angle milling head under typical working conditions are extracted in this study. The modal analysis results at an angle of 0° are shown in Figure 6 and Table 3.

From the analysis results in Figure 6 and Table 3, it can be seen that the first-order modal vibration mode of the swing angle milling head is mainly represented by the overall rotation and swing of the cardan frame. The maximum deformation is concentrated on the single side of the lower end of the cardan frame and the A-axis area. The natural frequency is 84.32 Hz, and the deformation is relatively large. The maximum deformation value is 555.39 μm. The second-order vibration mode is mainly manifested as the integral swing dominated by the cardan frame. The maximum deformation is concentrated on both sides of the lower end of the cardan frame and the A-axis area. The natural frequency is 89.14 Hz. The analysis shows that the initial design of the cardan frame structure is reasonable, and the rigidity of the upper part of the cardan frame is good, so the topological optimization can be further studied.

The static and modal analyses show that the structure of the swing angle milling head gimbal will affect the machining accuracy of the five-axis CNC planer milling machine, Therefore, it is necessary to optimize and improve its structure. On the premise that the stiffness and strength of the gimbal meet the design requirements, reduce the quality of the gimbal, which is beneficial to the improvement of the dynamic characteristics of the whole machine.

## 4. Optimization Design of Universal Carriage

Topology optimization is a powerful tool for searching for lightweight structures. This process can transform the optimal topology problem into an exploration and optimization of the material distribution problem in a designated design space [27,28] and can also maximize economic benefits. This article selects the maximum rigidity of the universal joint as the optimization objective, taking volume as the constraint condition, and integrates the theoretical foundation of the SIMP variable density method. The mathematical model for topology optimization is expressed as follows:

(3) $$\left\{\begin{array}{c} \min\;C=U^{T}KU=\sum_{e=1}^{N}u^{e}k^{e}u^{e}\\ s.t\;V=fV_{0}=\sum_{e=1}^{N}X_{e}V_{e}\leq V^{*}\\ F=KU\;\left(0<x_{\min}\leq x^{e}\leq x_{\max}\right) \end{array}\right.$$

where *C* is the objective function (overall flexibility of the universal joint); *F* is the force vector; *U* is the displacement matrix of the universal joint; *K* is the total stiffness matrix of the universal joint structure; *V*_0_ is the initial volume function; *f* is the optimized volume ratio; *V* is the optimized volume function; *V_e_* is the optimized volume of the gimbal unit; *X_e_* is the design variable (unit relative density); *x*_min_ and *x*_max_ are the minimum and maximum limit values of unit relative density; and *u^e^* is the unit displacement column vector.

### 4.1. Topology Optimization and Model Reconstruction of Universal Frame

**Figure 7:**
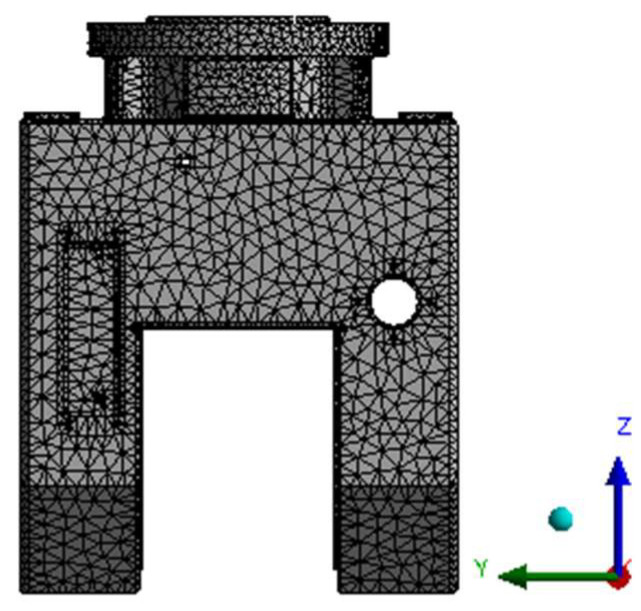
Mesh model of the universal joint.

**Figure 8:**
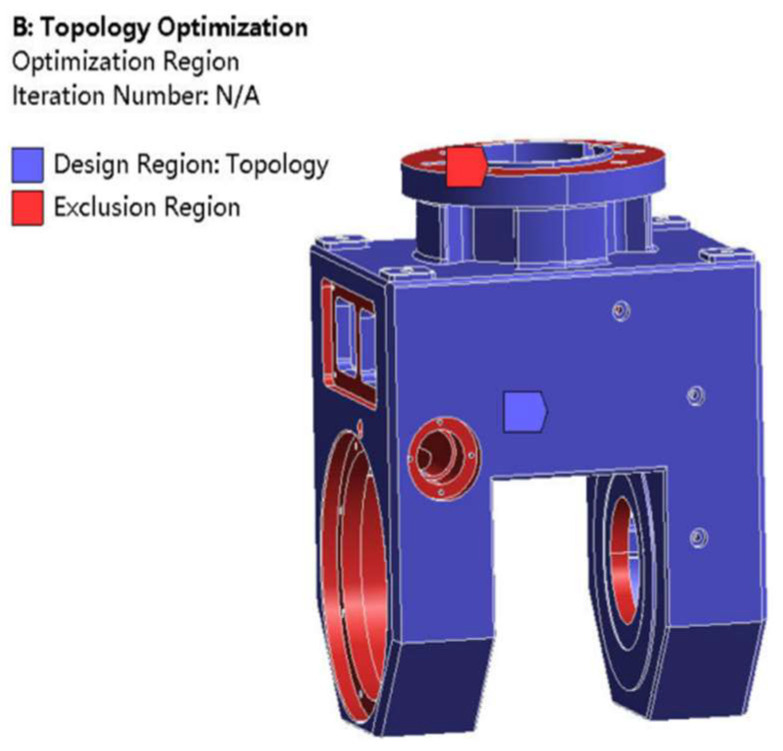
Schematic diagram of optimized and non-optimized areas for the gimbal.

After applying constraints and loads to the gimbal model for initial static analysis, the topology optimization module is used to optimize the gimbal’s topology. During the optimization process, the material of the universal joint is defined as ductile iron QT600-3, with a density of 7.12 g/cm^3^, a Poisson’s ratio of 0.285 and an elastic modulus of 158 GPa. The universal joint model is divided using the automatic grid division method, with a unit size of 10 mm. The grid model is shown in Figure 7. In order to avoid interference with the assembly of other structural components due to changes in the optimized structure of the universal joint, relevant areas where other components are installed on the universal joint and the areas on both sides of the universal joint are designated as non-optimized areas, while the remaining areas are designated as optimization objects. Figure 8 shows the division of the universal joint optimization area, with red indicating the exclusion area and blue indicating the optimization area.

**Figure 9:**
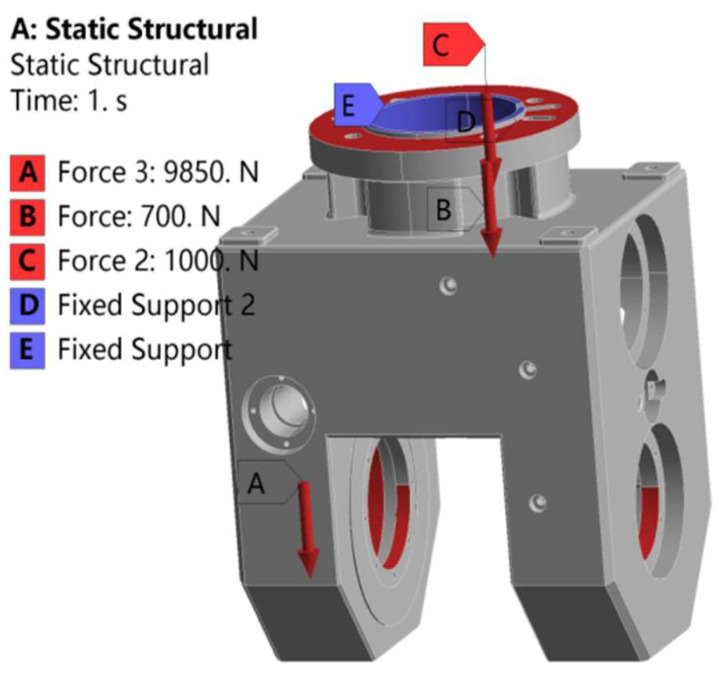
Load and constraint diagram of universal joint.

**Figure 10:**
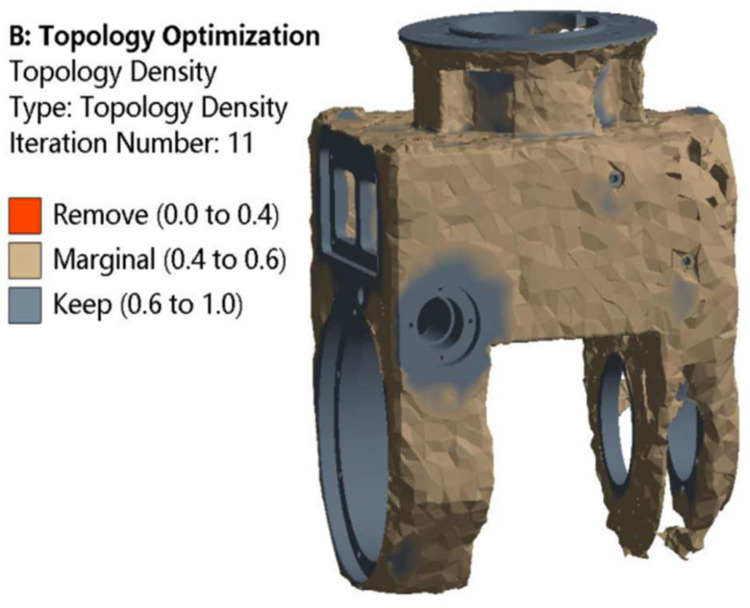
Density cloud map after optimizing the universal joint frame.

The constraints and loads imposed by the universal joint are shown in Figure 9. Constraints are added at the upper end of the universal joint, and the two toroidal surfaces in the middle of the upper end are subjected to a positive pressure of 700 N and 1000 N, respectively. A total of 9850 N of vertical downward pressure is applied to the four semicircular surfaces at the lower end. The material removal rate was set at 20% as the optimization objective for calculation, and after 11 iterations, the density cloud map of the optimized universal joint was obtained, as shown in Figure 10.

From Figure 10, it can be seen that the removal of material from the universal joint is mainly distributed at the upper and lower ends of both sides of the universal joint. Due to its irregular shape at the bottom and because the most important structural materials were retained, it can be seen from the static characteristic cloud diagram in the previous section that the maximum deformation is seen at the connection of the milling head and the tool handle, while the deformation at the bottom ends of both sides of the universal joint is relatively small. Therefore, the trapezoidal transition method at the bottom ends of both sides of the universal joint can be changed to arc transitions. At the same time, some material can also be removed from the area at the upper end of the universal joint. According to the modal analysis results in the previous section, different types of rib plates are considered in the design to achieve the design purpose due to the good rigidity of this area. Based on the above analysis, the optimization results of the swing angle milling head directly confirm the accuracy of the previous static mechanics and modal analysis results and can provide a reference for subsequent reconstruction design.

**Figure 11:**
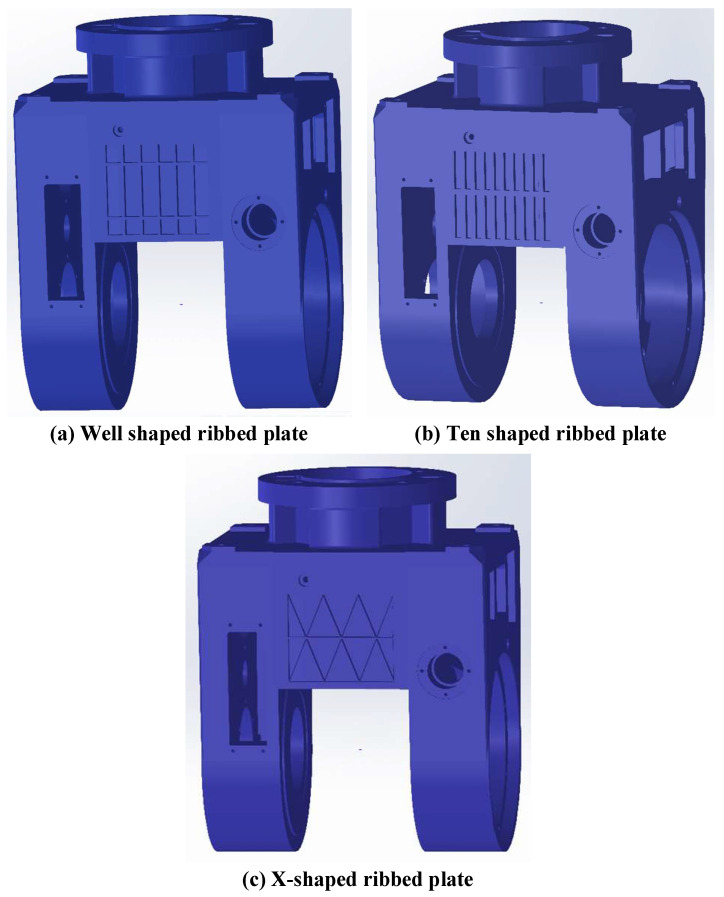
Universal frames with different rib plate configurations.

In order to reduce the quality of the universal joint and better align with the design requirements in terms of stiffness and strength, the materials on the upper sides of both ends of the universal joint are modified and redesigned based on the topology optimization results, resulting in a manufactured and assembled universal joint model. When designing, it is usually necessary to pay attention to the following two points. First, due to the different layout forms of the reinforcement plates, they will play different roles. For example, transverse reinforcement plates will have a bending resistance effect, and longitudinal reinforcement plates will have a torsional resistance effect. Therefore, selecting the appropriate reinforcement plate is particularly crucial. Second, the complexity of the rib plate structure and whether it is conducive to production and other factors must be fully considered. In response to these concerns, this article selects three commonly used rib plate layouts as the rib plate layouts on both sides of the universal joint. Figure 11 shows the universal joint with a “well”-shaped rib plate, “ten”-shaped rib plate and “X”-shaped rib plate configuration, respectively.

**Figure 12:**
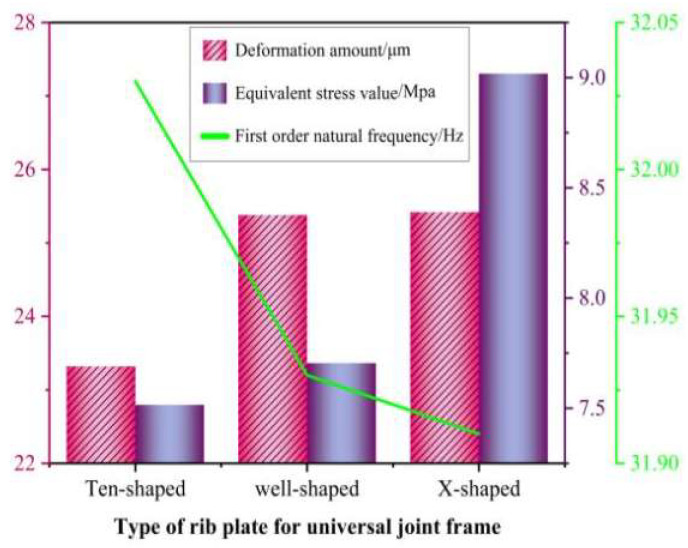
Static and modal analysis results of three types of rib plate configurations of universal frames.

The static and modal analysis results of the three types of rib plate configurations of the universal joint were obtained through finite-element analysis, as shown in Figure 12. From Figure 12, it can be seen that the minimum deformation and equivalent stress among the three rib plate configurations of the universal joint is the “ten”-shaped rib plate. Compared with the other two rib plate structures, the “ten”-shaped rib plate configuration has the best static stiffness in the universal joint. Moreover, compared to the other two rib plate configurations, the ten-shaped rib plate configuration of the universal joint has the highest first-order natural frequency and the best dynamic stiffness. Analysis shows that the ten-shaped rib plate configuration of the universal joint has a better static and dynamic performance than the other two rib plate configurations. Therefore, this article uses the ten-shaped rib plate structure to replace the materials on both sides of the original universal joint.

### 4.2. Performance Analysis of Optimized Universal Joint Model

**Figure 13:**
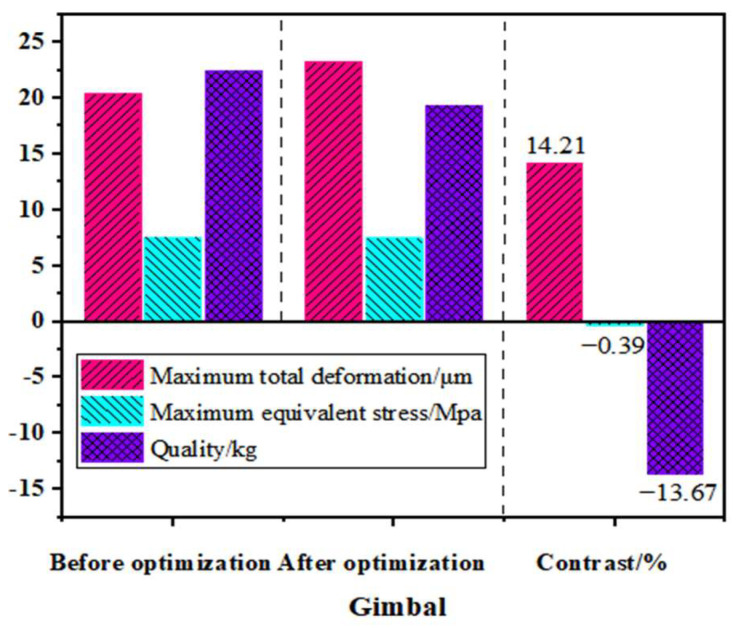
Comparison of static characteristics and mass before and after optimization of universal joint frame.

The reconstructed universal joint model was analyzed using Workbench finite-element simulation software, and its stress–strain, mass and other parameters are shown in Figure 13. From Figure 13, it can be seen that the total deformation of the optimized universal joint increased by 14.21%, which meets the precision requirements of the swing angle milling head and does not have a significant impact on the performance of the entire machine. The equivalent stress decreased by 0.39%, and the mass decreased from 22.45 kg to 19.38 kg, with a weight reduction ratio of 13.67%. The optimization of the design was achieved, and the effect was significant.

**Figure 14:**
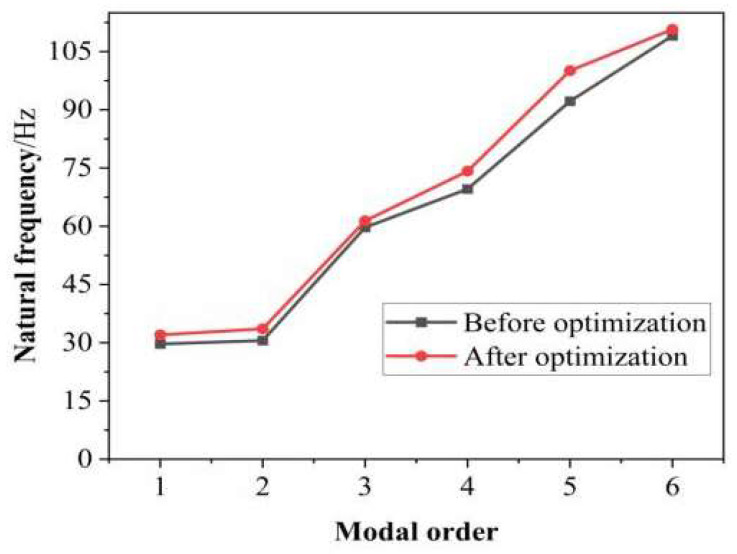
Comparison diagram of the first 6 natural frequencies of the optimized front and rear universal joint.

The same material properties and constraints as the original model were loaded for modal analysis, and the first six natural frequencies of the universal joint model with a “ten” rib plate configuration were recorded. The natural frequencies of the universal joint model before and after optimization are shown in Figure 14.

From Figure 14, it can be seen that the natural frequency of the optimized swing angle milling head universal joint increases to varying degrees. The natural frequencies of the milling head universal joint from the first to sixth orders have been increased by 7.9%, 9.92%, 2.81%, 6.18%, 8.53% and 1.54%, respectively. Among them, the fundamental frequency of the universal joint is significantly increased, reducing the probability of resonance. The first natural frequency of the optimized universal joint is 32.03 Hz, which is higher than the 25 Hz corresponding to the highest speed of the mechanical swing angle milling head under normal working conditions. This further indicates that the improved universal joint’s natural frequency will not affect the normal operation of the swing angle milling head.

**Figure 15:**
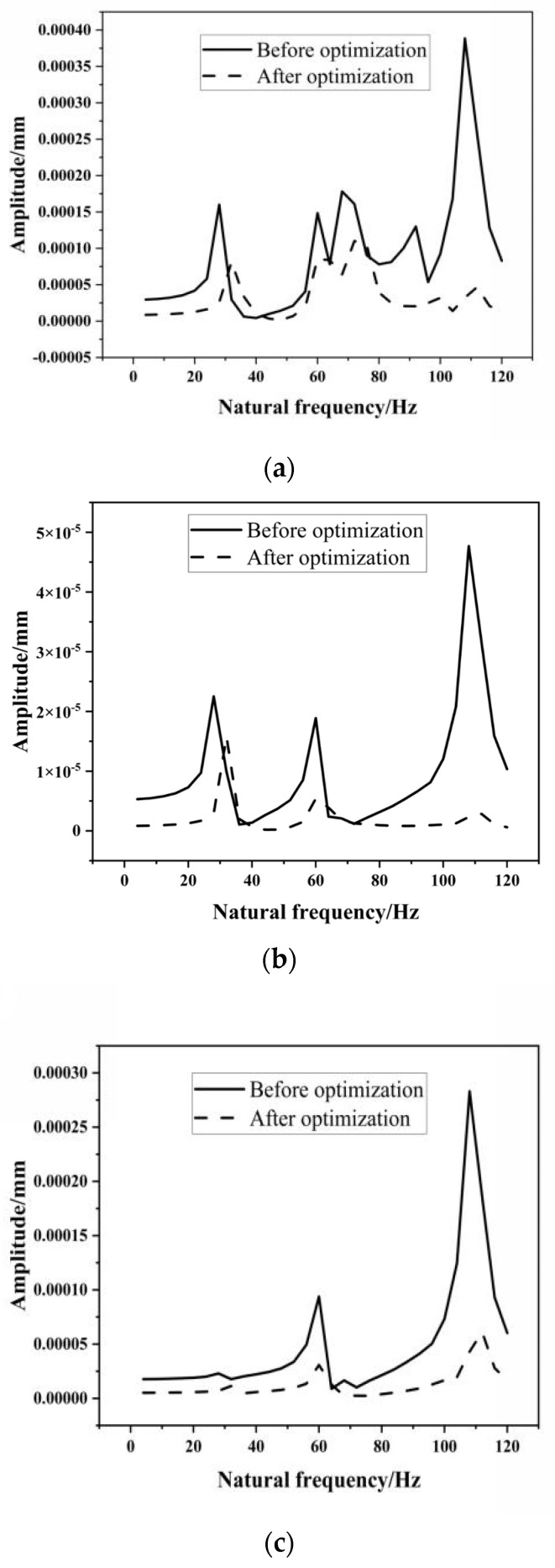
Comparison of response harmonic lines of the universal carriage: (**a**) X direction, (**b**) Y direction, (**c**) Z direction.

Figure 15 shows the harmonic response analysis results of the universal joint in all directions before and after optimization. By comparing the response spectral lines before and after optimization, it can be seen that the frequencies at the peak points of the universal joint in the X, Y and Z directions are basically consistent with the first six modal results, which indirectly verifies the accuracy of the modal analysis. The peak values corresponding to the fundamental frequency in the X and Y directions are significantly reduced. The Y direction is relatively stable in the frequency range of 70–100 Hz, while the Z direction is relatively stable in the frequency range of 0–50 Hz. At the same time, a backward shift in the peak positions occurs to varying degrees in the three directions, which indicates a significant improvement in the dynamic performance of the universal joint and verifies the rationality of the optimization results of the universal joint.

## 5. Conclusions and Prospect

In order to reduce the mass of the swing angle milling head and improve the dynamic characteristics of the whole machine, the mechanical characteristics of the swing angle milling head of a heavy-duty CNC milling machine are studied in this paper. Based on the cloud chart of the static and dynamic characteristics, it can be seen that the gimbal structure with the largest proportion of the mass of the milling head and the strongest rigidity is the research object to be optimized. The gimbal is then optimized and improved using the topology optimization method. The following conclusions can be drawn:

(1) By analyzing the static and dynamic performance of the gimbal with different stiffened plate structures, combined with the optimization results, the “ten”-type stiffened plate structure with the best performance is finally selected as the optimized structure of the upper end of the gimbal.
(2) Through the analysis of the results of the gimbal before and after optimization, it is shown that, under the premise that the stiffness and strength meet the design requirements, the weight reduction ratio of the optimized gimbal structure is 13.67%, and the weight reduction effect is obvious.
(3) After optimization, the first six natural frequencies of the gimbal are increased, among which the first natural frequency is increased by 7.9%, which can effectively avoid resonance. After optimization, the maximum response amplitudes of the gimbal in the X, Y and Z directions are reduced, which is conducive to the improvement of the dynamic performance of the whole machine.

Since this paper only studies the gimbal with the greatest mass and the highest stiffness in the swing angle milling head, in-depth topology optimization research will be carried out for other key components of the swing angle milling head to achieve the optimal mechanical properties of the entire milling head. In addition, the optimization method proposed in this paper provides a theoretical basis and reference for the lightweight research of other key components (such as the beam, spindle box, column, etc.) in similar machine tools. This research provides an important theoretical reference and methodological support for improving the static and dynamic characteristics of machine tools.

## Figures and Tables

**Table 1:** Specific parameters of materials used for milling head simulation.

Material Properties	38CrMoAl	QT600-3	Alloy Structural Steel
Elastic modulus E (GPa)	209	158	206
Poisson’s ratio (μ)	0.295	0.285	0.3
Density (kg/m^3^)	7266	7120	7850

**Table 2:** Table of milling parameters for the swing angle milling head.

Name	*f_z_* (mm)	*a_e_* (mm)	*a_p_* (mm)	*C_FC_*	*D* (mm)	*Z*
Parameter value	0.2	35	2	773	60	4

**Table 3:** The first two modal characteristics of the milling head at an angle of 0°.

Order	Natural Frequency/Hz	Description of Milling Head Vibration Mode
1	84.32	The milling head mainly composed of a universal gimbal rotates along the Z-axis and swings along the X-axis
2	89.14	The middle and lower ends of the milling head based on a universal gimbal swing left and right along the Y-axis

## Data Availability

All data included in this study are available upon request by contact with the corresponding author.
